# Supplementary material for: Organization of the human intestine at single-cell resolution
Source: Nature. 2023 Jul 19;619(7970):572–84. doi: 10.1038/s41586-023-05915-x (PMC10356619; doi:10.1038/s41586-023-05915-x)
Supplement: Supplementary file 1 — Additional discussion of the results. [file 41586_2023_5915_MOESM1_ESM.pdf]

nature portfolio

https://doi.org/10.1038/s41586-023-05915-x

Supplementary information

Organization of the human intestine at single-cell resolution

In the format provided by the authors and unedited

nature portfolio

https://doi.org/10.1038/s41586-023-05915-x

Supplementary information

Organization of the human intestine at single-cell resolution

In the format provided by the authors and unedited

Supplementary Discussion

Aberrations in cellular density have been associated with disease states such as inflammatory bowel disease (IBD). As the intestine is the largest immune organ in the body[21], evaluating immune cell localization and cellular interactions in the gut is critical for understanding oral vaccine design[22], interactions regulating the gut microbiota[23], regulation of allergic food responses[24], axes critical for wound repair[25], and immune system responses and immunotherapies for cancer[26–29]. We observed that plasma cells have one of the highest same-cell densities within the intestine. Plasma cells require a special niche for survival in the bone marrow[85] with survival factors such as CD44, a proliferation-inducing ligand (APRIL), IL-6, and SDF-1[85,86]. Localization of plasma cells is critical in gut-associated lymphoid tissue, playing an important role in immunity by secreting IgA antibodies, the most abundantly produced antibody, critical for maintaining a homeostatic relationship with microbiota and food antigens[83,84]. We found the *plasma cell enriched* neighborhood form an important crossroads of several other immune and epithelial neighborhoods within the mucosa. We also found plasma cells colocalized with antigen-presenting cells, sources of APRIL, which drives ectopic germinal center formation and plasma cell infiltration in cases of inflammation[32]. Collectively, these observations suggest a role for antigen-presenting cells in curating a subepithelial niche for plasma cells in the intestine in addition to transit amplifying epithelial cells.

CD8+ T cells are critical for antiviral cytotoxic function and were one of the few cell types defined within the CODEX data shown to decrease from the small intestine to the colon. This compositional change likely reflects differences in antigen availability from both initial exposure as well as greater access to food antigens and foreign material with less mucus-secreting cells as compared to the colon[33,88] that may also be a cause for increased DCs we see within the colon. Dysregulation of CD8+ T IEL cells is associated with IBD and celiac disease. And the identification of CD8+ T IEL neighborhood further confirms the phenotypic distinction of IEL CD8+ T cells from the rest of CD8+ T cells within the intestine and merits additional study to understand maintenance, regulation, and renewal[89] of these cells. Finally, studying changes in T cell percentages within the gut in patients with hypertension may provide cell type mechanisms for associations of hypertension with other diseases such as colon cancer[93,94]. Indeed, CD8+ T cells presence and density correlate with beneficial anti-cancer outcomes[90] and cancer rates are increased within the colon as compared to the small intestine[91,92]. It will be interesting to explore this correlation in the future and understand if intraepithelial immune cells help prune defective epithelial cells.

CD4+ T cells are involved both in CD4+ T cell support of B cell and CD8+ T cell activation. We found that CD4+ T cells were broadly distributed and enriched in all immune-rich multicellular structures (*innate immune enriched, inner follicle, outer follicle, adaptive immune enriched,* and

plasma cell enriched) characteristic of their supportive functions. Consequently, the broad involvement of CD4+ T cells in diverse immune multicellular structures suggests their modulation might be akey therapeutic target for regulating immune responses in the intestine. Integration of CODEX and snRNAseq at the single-cell level enabled analysis of the *inner follicle* neighborhood gene expression where we see differential expression of genes involved in active immune responses. Further understanding gene expression by location and validation with spatial transcriptomics will be important to clarify functional differences, development, and maintenance, as follicles are associated with beneficial cancer outcomes, increased autoimmunity, and clearance of infection[10,87].

Interestingly, we are able to find major structures of the intestine by common neighborhood analysis, but also are able to identify differences in the cellular makeup of these neighborhoods. We investigated this more broadly to see which neighborhoods and cell types within these neighborhoods were conserved as defined by common cell type enrichment scores between the small and large intestine. One of the cell neighborhoods that were most differential were the intestinal crypt cellular neighborhoods. We also observed CD4+ T cells and CD8+ T cells enriched at the base of the crypt with the stem cell zones of the small intestine and colon. Understanding the colocalization with the crypt, interactions with stem cells, and signaling to other stromal cells will be important to learn how the role these cells play in this curated microenvironment.

We generated a spatially hierarchical description of the intestine that is derived from the cell type labels we generate in our CODEX multiplexed imaging data. The first level we computed was the immediate cellular neighborhoods within the tissues by examining conserved cell type composition within ten nearest neighbors of cells[10]. These represented and identified microstructures found within the intestine such as the vasculature or immune follicles. We further built upon the neighborhood concept to understand how neighborhoods of cells are interacting with each other and tend to colocalize. We quantitatively characterized these within *communities* of multicellular structures which can be further categorized into major *tissue units*. This categorization isolated mucosal areas of the tissue enriched with immune and epithelial cells and defined overall structures and interactions of multicellular neighborhoods in comparing the colon and small intestine. Using this hierarchical description of the intestine we were able to capture the spatial layering of the intestine from the muscularis mucosa to the lumen of the intestine.

Leveraging paired transcriptome and chromatin accessibility data, we achieved further granularity to define the diversity of cell types in the intestine. Overall, different regions of the colon exhibited highly concordant cell type abundances. However, the cell type compositions of

the small intestine regions were more diverse as compared to the colon, with the ileum often exhibiting immune and stromal cell type fractions shared between the small intestine regions and the colon. We observe greater diversity of specialized epithelial cells, including enteroendocrine and mucin-secreting cells in the small intestine. Within all regions, we identified goblet cells with high expression of MUC2. However, in the duodenum we identified an additional cluster containing cells characterized by high expression of the gel forming mucin, MUC6. The MUC6 cluster may represent cells of the duodenal Brunner's glands, which have been shown to express high levels of MUC6[51].

We also further improved our analysis of the data by integrating the molecular detail of the snRNAseq and the spatial detail of the CODEX multiplexed imaging datasets at the single cell level. While various computational tools are available to integrate single cells across sequencing modalities[49–51], integration involving protein modalities, especially spatial-proteo modalities like CODEX, remain challenging. This is mostly due to the fact that protein modalities have limited shared features and therefore is an information bottleneck (~30-60 shared features vs >1000 available across sequencing modalities). To solve this, we implemented with MaxFuse (manuscript in preparation), a method we specifically designed towards such challenging integration tasks: linear assignment coupled with graph smoothing and meta cell construction. This works on both weakly correlated-shared features and non-shared features and was used to generate single-cell level pairing information across CODEX and snRNAseq datasets that allowed us to evaluate differential gene expression across cellular neighborhoods.

This work follows and extends the work of several scRNA gut atlases, including an atlas of the small intestine in mice (Nature 2017) and two recent atlases describing the small and large intestine in humans (Nature 2021, Cellular and Molecular Gastroenterology and Hepatology 2022). The first of these human studies additionally provides information on intestinal development in addition to mapping cell types and features of the adult human intestine. Our work supports many of the findings in these previously published atlases. For example, the two recent human gut atlases both identified Best4+ enterocytes, first identified in the colon (Smillie et al 2019), in human small intestine and large intestine, which we observe in our data as well.

Chromatin accessibility data also allow assessment of the cell-type specific enrichment with regulatory elements of common genetic variation linked to prevalent intestinal diseases (e.g. GWAS hits) such as Celiac, ulcerative colitis, and Crohn's disease. This analysis can nominate the specific cell types through which these GWAS hits may be functioning, as well as the cell types that may be driving disease etiology. We find that T cells are most enriched in the autoimmune conditions, Celiac disease, ulcerative colitis, and Crohn's. We also find that heritability of BMI is

enriched in enteroendocrine cells, suggesting that variation in BMI is likely partially driven by genetic variation in regions that are functional in intestine enteroendocrine cells. Regardless, overall we can assign heritability of specific diseases to specific intestinal cell types.
