# Supplementary material for: Organization of the human intestine at single-cell resolution
Source: Nature. 2023 Jul 19;619(7970):572–84. doi: 10.1038/s41586-023-05915-x (PMC10356619; doi:10.1038/s41586-023-05915-x)
Supplement: Supplementary file 2 — Reporting Summary [file 41586_2023_5915_MOESM2_ESM.pdf]

nature portfolio

Corresponding author(s): Garry Nolan, William Greenleaf, Mike Snyder

Last updated by author(s): 10/28/2022

Reporting Summary

Nature Portfolio wishes to improve the reproducibility of the work that we publish. This form provides structure for consistency and transparency in reporting. For further information on Nature Portfolio policies, see our Editorial Policies and the Editorial Policy Checklist.

Statistics

For all statistical analyses, confirm that the following items are present in the figure legend, table legend, main text, or Methods section.

n/a	Confirmed	
☐	☒	The exact sample size (*n*) for each experimental group/condition, given as a discrete number and unit of measurement
☐	☒	A statement on whether measurements were taken from distinct samples or whether the same sample was measured repeatedly
☐	☒	The statistical test(s) used AND whether they are one- or two-sided<br>*Only common tests should be described solely by name; describe more complex techniques in the Methods section.*
☒	☐	A description of all covariates tested
☐	☒	A description of any assumptions or corrections, such as tests of normality and adjustment for multiple comparisons
☐	☒	A full description of the statistical parameters including central tendency (e.g. means) or other basic estimates (e.g. regression coefficient) AND variation (e.g. standard deviation) or associated estimates of uncertainty (e.g. confidence intervals)
☐	☒	For null hypothesis testing, the test statistic (e.g. *F*, *t*, *r*) with confidence intervals, effect sizes, degrees of freedom and *P* value noted<br>*Give P values as exact values whenever suitable.*
☒	☐	For Bayesian analysis, information on the choice of priors and Markov chain Monte Carlo settings
☒	☐	For hierarchical and complex designs, identification of the appropriate level for tests and full reporting of outcomes
☐	☒	Estimates of effect sizes (e.g. Cohen's *d*, Pearson's *r*), indicating how they were calculated

Our web collection on statistics for biologists contains articles on many of the points above.

Software and code

Policy information about availability of computer code

Data collection	Code for generating fragments files for scATAC and counts matricies for single cell RNA was obtained from 10x genomics (go.10xgenomics.com/scATAC/cell-ranger-ATAC and https://support.10xgenomics.com/single-cell-gene-expression/software/pipelines/latest/what-is-cell-ranger). Versions for processing of the singleome datasets were cellranger-3.1.0 and cellranger-atac-1.2.0. cellranger-arc-1.0.1 was used to process the multiome samples.<br>And code for processing CODEX multiplexed imaging data (https://github.com/nolanlab/CODEX)
Data analysis	macs2 2.1.1.20160309 – Software for peak calling<br>R version 4.1.2 – R environment for analysis of single cell data<br>ArchR - 1.0.1 - Software for analysis of snATAC-seq data.<br>Seurat_4.1.0 - Software for analysis of snRNA-seq data.<br>DoubletFinder_2.0.3 – Software for doublet removal for scRNA-seq<br>BSgenome.Hsapiens.UCSC.hg38_1.4.3 – Package containing genomic DNA sequences<br>harmony_0.1.0 - R package used for integration of single-cell data.<br>ggplot2_3.3.5 - R package used for plotting of single cell data.<br>miloR_1.2.0 - R package for analysis of differential abundance.<br><br>Python 3.9.0 - Python version for scCODA analysis<br>sccoda version 0.1.8 - Python package for analysis of differential abundance.<br>scanpy Python package (version 1.9.1) - analyzing single cell CODEX data<br>R version 4.2.0 – R version for GO enrichment analysis<br>limma_3.52.2 – Software used for GO enrichments<br><br>R version 4.0.2 – R environment for initial analysis of single-cell data<br>Seurat_4.0.1 – Software for initial analysis of snRNA-seq data.

Custom code for analyzing the snATAC and snRNA data is available on GitHub (https://github.com/winstonbecker/scColonHuBMAP).

Code for clustering (https://github.com/nolanlab/vortex), the code for transferring cell type labels with STELLAR (https://github.com/snap-stanford/stellar), code for neighborhood analysis (https://github.com/nolanlab/NeighborhoodCoordination), and code for tissue schematics (https://github.com/nolanlab/TissueSchematics) is available on github.

For manuscripts utilizing custom algorithms or software that are central to the research but not yet described in published literature, software must be made available to editors and reviewers. We strongly encourage code deposition in a community repository (e.g. GitHub). See the Nature Portfolio guidelines for submitting code & software for further information.

Data

Policy information about availability of data

All manuscripts must include a data availability statement. This statement should provide the following information, where applicable:

- Accession codes, unique identifiers, or web links for publicly available datasets
- A description of any restrictions on data availability
- For clinical datasets or third party data, please ensure that the statement adheres to our policy

All of the published datasets in this study can be visualized and assessed through a website portal (https://portal.hubmapconsortium.org/). We have created a landing page with links to all the raw dataset IDs and the HuBMAP ID for this Collection is HBM692.JRZB.356 and the DOI is:10.35079/HBM692.JRZB.356. Supplemental Table 10 also lists all the dataset IDs within the HuBMAP portal where all raw datasets are stored that can be downloaded and also viewed in a processed state. We also provide the processed and annotated single-cell CODEX datasets with labeled cell types, neighborhoods, communities, tissue units, and also protein expression via Dryad doi https://doi.org/10.5061/dryad.pk0p2ngrf.

Field-specific reporting

Please select the one below that is the best fit for your research. If you are not sure, read the appropriate sections before making your selection.

☒ Life sciences ☐ Behavioural & social sciences ☐ Ecological, evolutionary & environmental sciences

For a reference copy of the document with all sections, see nature.com/documents/nr-reporting-summary-flat.pdf

Life sciences study design

All studies must disclose on these points even when the disclosure is negative.

Sample size	Sample sizes were set to maximize diversity across 8 regions of the intestine while also capturing sufficient depth in single-cell techniques that are able to capture differences in composition between cell types at resolution using multiome and CODEX multiplexed imaging modalities.
Data exclusions	All datasets generated that did not fail experimentally (e.g. overloaded sample) were included in the study.
Replication	Experimental assays were not replicated in this study, but the same measurements were made for all 9 donors at all 8 regions of the intestine. For CODEX multiplexed imaging 8 of the 9 donors included the full panel and were used for generating main figure analysis. snRNAseq and snATACseq were completed on 3/9 samples and multiome analysis was completed on 6/9 samples.
Randomization	Randomization was not relevant for this study as there were not multiple groups requiring randomization.
Blinding	No blinding was performed in this study because there were no experimental groups.

Reporting for specific materials, systems and methods

We require information from authors about some types of materials, experimental systems and methods used in many studies. Here, indicate whether each material, system or method listed is relevant to your study. If you are not sure if a list item applies to your research, read the appropriate section before selecting a response.

Materials & experimental systems

n/a	Involved in the study	
☐	☒	Antibodies
☒	☐	Eukaryotic cell lines
☒	☐	Palaeontology and archaeology
☒	☐	Animals and other organisms
☐	☒	Human research participants
☒	☐	Clinical data
☒	☐	Dual use research of concern

Methods

n/a	Involved in the study	
☒	☐	ChIP-seq
☒	☐	Flow cytometry
☒	☐	MRI-based neuroimaging

Antibodies

Antibodies used

We provide a detailed antibody information and metadata for all the antibodies used for CODEX (>60) within Supplementary Table 7 of the submission. Here is it below:
antibody_name rr_id uniprot_accession_number lot_number dilution conjugated_cat_number conjugated_tag Validation
Anti-MUC-2 antibody AB_791261 Q02817 7/5/19 1/200 custom 63-Alexa 488 validated for IHC/IF by manufacturer
Anti-MUC-1/EMA antibody AB_2864392 P15941 10/7/20 1/100 custom 15-Alexa 488 validated for IHC/IF by manufacturer
Anti-Synaptophysin antibody AB_10010435 P08247 1/22/20 1/100 custom 69-Alexa 488 validated for IHC/IF by manufacturer
Anti-CD15 antibody AB_395800 P22083 11/2/20 1/200 custom 70-Alexa 488 validated on fresh frozen human lymphoid tissue; Eur. J. Immunol. 2021
Anti-ITLN1 antibody AB_2129678 Q8WWA0 1/22/20 1/200 custom 72-Alexa 488 validated for IHC/IF by manufacturer
Anti-Vimentin antibody AB_393716 P08670 3/20/18 1/200 custom 7-Alexa 488 validated on fresh frozen human lymphoid tissue; Eur. J. Immunol. 2021
Anti-CD11c antibody AB_395792 P20702 1/22/18 1/200 custom 44-Alexa 488 validated in lab with fresh frozen human intestine tissue with negative and positive controls
Anti-BCL-2 antibody AB_2864404 P10415 7/30/19 1/200 custom 41-Alexa 488 validated for IHC/IF by manufacturer
Anti-CD38 antibody AB_2561794 P28907 4/13/18 1/500 custom 66-Alexa 488 validated on fresh frozen human lymphoid tissue; Eur. J. Immunol. 2021
Anti-a-SMA antibody AB_2572996 P62736 9/10/19 1/200 custom 8-Alexa 488 validated for IHC/IF by manufacturer
Anti-CD66 antibody AB_394166 P13688 3/20/18 1/200 custom 5-Alexa 488 validated on fresh frozen human lymphoid tissue; Eur. J. Immunol. 2021
Anti-CD68 antibody AB_1089058 P34810 8/12/19 1/100 custom 48-Alexa 488 validated for IHC/IF by manufacturer
Anti-CD7 antibody AB_1659214 P09564 3/23/18 1/200 custom 58-Alexa 488 validated on fresh frozen human lymphoid tissue; Eur. J. Immunol. 2021
Anti-CD45RO antibody AB_314418 P08575 8/12/19 1/100 custom 36-Alexa 488 validated for IHC/IF by manufacturer
Anti-Collagen IV antibody AB_305584 P02462 4/13/18 1/100 custom 33-Alexa 488 validated on fresh frozen human lymphoid tissue; Eur. J. Immunol. 2021
Anti-SOX9 antibody AB_2665492 P48436 7/5/19 1/100 custom 26-Cy3 validated for IHC/IF by manufacturer
Anti-GATA-3 antibody AB_2108590 P23771 3/23/18 1/100 custom 55-Cy3 validated for IHC/IF by manufacturer
Anti-Lefty antibody AB_2797977 O00292 2/26/20 1/100 custom 71-Cy3 validated in lab with fresh frozen human intestine tissue with negative and positive controls
Anti-CHGA antibody AB_2864388 P10645 7/25/19 1/100 custom 2-Cy3 validated for IHC/IF by manufacturer
Anti-CD4 antibody AB_2561907 P01730 1/22/18 1/200 custom 28-Cy3 validated on fresh frozen human lymphoid tissue; Eur. J. Immunol. 2021
Anti-HLA-DR antibody AB_2562826 P04233 7/24/18 1/200 custom 11-Cy3 validated on fresh frozen human lymphoid tissue; Eur. J. Immunol. 2021
Anti-CD44 antibody AB_312953 P16070 1/22/20 1/200 custom 14-Cy3 validated for IHC/IF by manufacturer
Anti-CD3 antibody AB_314056 P07766 3/23/18 1/200 custom 20-Cy3 validated on fresh frozen human lymphoid tissue; Eur. J. Immunol. 2021
Anti-CD90 antibody AB_940393 P04216 3/23/18 1/200 custom 68-Cy3 validated on fresh frozen human lymphoid tissue; Eur. J. Immunol. 2021
Anti-CD21 antibody AB_11219188 P20023 1/22/18 1/500 custom 21-Cy3 validated on fresh frozen human lymphoid tissue; Eur. J. Immunol. 2021
Anti-CD57 antibody AB_535988 Q9P2W7 2/22/18 1/100 custom 30-Cy3 validated on fresh frozen human lymphoid tissue; Eur. J. Immunol. 2021
Anti-CD34 antibody AB_1732014 P28906 12/8/19 1/100 custom 80-Cy3 validated on fresh frozen human lymphoid tissue; Eur. J. Immunol. 2021
Anti-CD36 antibody AB_395846 P16671 2/13/18 1/200 custom 49-Cy3 validated in lab with fresh frozen human intestine tissue with negative and positive controls
Anti-Cytokeratin antibody AB_2616960 Q04695 2/22/18 1/200 custom 67-Cy3 validated on fresh frozen human lymphoid tissue; Eur. J. Immunol. 2021
Anti-CD117 antibody AB_2131466 P10721 2/9/18 1/200 custom 74-Cy3 validated on fresh frozen human lymphoid tissue; Eur. J. Immunol. 2021
Anti-CD19 antibody AB_395810 P15391 3/13/20 1/200 custom 75-Cy3 validated on fresh frozen human lymphoid tissue; Eur. J. Immunol. 2021
Anti-CD45 antibody AB_314390 P08575 5/21/18 1/500 custom 56-Cy3 validated on fresh frozen human lymphoid tissue; Eur. J. Immunol. 2021
Anti-CD69 antibody AB_314837 Q07108 4/13/18 1/500 custom 24-Cy3 validated for IHC/IF by manufacturer
Anti-Somatostatin antibody AB_2890053 P61278 2/3/20 1/100 custom 57-Cy3 validated for IHC/IF by manufacturer
Anti-CD49a antibody AB_1236385 P56199 2/17/20 1/50 custom 46-Cy3 validated for IHC/IF by manufacturer
Anti-CD161 antibody AB_1501090 Q12918 1/22/20 1/200 custom 53-Cy3 validated for IHC/IF by manufacturer
Anti-MUC6 antibody AB_2864391 Q6W4X9 8/23/19 1/100 custom 81-Cy5 validated in lab with fresh frozen human intestine tissue with negative and positive controls
Anti-CD31 antibody AB_395837 P16284 2/22/18 1/200 custom 42-Cy5 validated on fresh frozen human lymphoid tissue; Eur. J. Immunol. 2021
Anti-CD49f antibody AB_2296273 P23229 3/23/18 1/50 custom 51-Cy5 validated on fresh frozen human lymphoid tissue; Eur. J. Immunol. 2021
Anti-CDX2 antibody AB_2864406 Q99626 11/22/19 1/200 custom 62-Cy5 validated for IHC/IF by manufacturer
Anti-CD127 antibody AB_10718513 P16871 2/13/18 1/100 custom 61-Cy5 validated for IHC/IF by manufacturer
Anti-CD8 antibody AB_1877104 P01732 10/7/20 1/200 custom 43-Cy5 validated on fresh frozen human lymphoid tissue; Eur. J. Immunol. 2021
Anti-CD16 antibody AB_395804 P08637 2/22/18 1/25 custom 52-Cy5 validated on fresh frozen human lymphoid tissue; Eur. J. Immunol. 2021
Anti-CD123 antibody AB_395455 P26951 2/13/18 1/25 custom 59-Cy5 validated on fresh frozen human lymphoid tissue; Eur. J.

Immunol. 2021
Anti-CD279 (PD-L1) antibody AB_2864409 Q15116 2/22/18 1/50 custom 79-Cy5 validated for IHC/IF by manufacturer
Anti-NKG2D (CD314) antibody AB_492956 P26718 8/29/19 1/100 custom 77-Cy5 validated for IHC/IF by manufacturer
Anti-CD206 antibody AB_571923 P22897 3/29/19 1/200 custom 25-Cy5 validated for IHC/IF by manufacturer
Anti-aDefensin 5 antibody AB_2864387 Q01523 8/30/19 1/200 custom 60-Cy5 validated for IHC/IF by manufacturer
Anti-CD138 antibody AB_2561790 P18827 3/29/19 1/100 custom 76-Cy5 validated for IHC/IF by manufacturer
Anti-CK7 antibody AB_2864389 P08729 7/25/19 1/200 custom 3-Cy5 validated for IHC/IF by manufacturer
Anti-PGP9.5 antibody AB_2890054 P09936 1/22/20 1/200 custom 23-Cy5 validated for IHC/IF by manufacturer
Anti-Podoplanin antibody AB_1595511 Q86YL7 4/13/18 1/100 custom 32-Cy5 validated on fresh frozen human lymphoid tissue; Eur. J. Immunol. 2021
Anti-CD56 antibody AB_395904 P13591 2/22/18 1/100 custom 29-Cy5 validated for IHC/IF by manufacturer
Anti-CD154 antibody AB_314825 P29965 8/22/18 1/200 custom 38-Cy5 validated for IHC/IF by manufacturer
Anti-Ki67 antibody AB_396287 P46013 4/13/18 1/25 custom 6-Cy5 validated on fresh frozen human lymphoid tissue; Eur. J. Immunol. 2021
Anti-CD163 antibody AB_1088991 Q86VB7 3/29/19 1/100 custom 45-Cy5 validated for IHC/IF by manufacturer
Anti-CD294 antibody AB_10639863 Q9Y5Y4 3/23/18 1/100 custom 65-Cy5 validated for IHC/IF by manufacturer
Anti-CD25 antibody AB_1107617 P01589 7/18/18 1/100 custom 57-Cy3 validated for IHC/IF by manufacturer
Anti-OLFM4 antibody AB_2785318 Q6UX06 10/7/20 1/100 custom 65-Cy3 validated for IHC/IF by manufacturer
Anti-Lysozyme antibody AB_776115 P61626 7/16/20 1/25 custom 81-Cy3 validated in lab with fresh frozen human intestine tissue with negative and positive controls
Anti-CD33 antibody AB_314342 P20138 10/7/20 1/100 custom 23-Cy3 validated for IHC/IF by manufacturer
Anti-FAP antibody AB_2532994 Q12884 2/22/20 1/25 custom 79-Cy5 validated for IHC/IF by manufacturer
Anti-CD98 antibody AB_2302070 P08195 7/16/20 1/25 custom 55-Cy5 validated for IHC/IF by manufacturer
Anti-CD147 antibody AB_314586 P35613 7/16/20 1/100 custom 71-Cy5 validated for IHC/IF by manufacturer

Validation

We provide a detailed antibody information and metadata for all the antibodies used for CODEX (>60) within Supplementary Table 7 and methods of validation from source vendor, prior publications, and within our primary data and in the above box.

Human research participants

Policy information about studies involving human research participants

Population characteristics

Individuals at Washington University in St. Louis were identified to participate in this study. We analyzed eight sections from nine individuals: seven European-ancestry (five males and two females), one African American male, and one African American female. Age ranges were from 24 to 78 years.

Recruitment

Patients without known intestinal diseases were recruited for this study. Participants were recruited for research after next-of-kin consented for organ donation. Given that presentation for organ donation after death is random, we do not expect biases to be present. Our experience is that the demographic composition of recruited participants largely reflects the composition of the local population (St. Louis, MO).

Ethics oversight

This study complies with all relevant ethical regulations and was approved by the Washington University Institutional Review Board and the Stanford University Institutional Review Board. Human bowel tissues were procured from deceased organ donors. Written informed consent was obtained from next-of-kin for all donor subjects.Participants were recruited for research after next-of-kin consented for organ donation. Given that presentation for organ donation after death is random, we do not expect biases to be present. Our experience is that the demographic composition of recruited participants largely reflects the composition of the local population (St. Louis, MO).

Note that full information on the approval of the study protocol must also be provided in the manuscript.

March 2021
