# Supplementary material for: Organization of the human intestine at single-cell resolution
Source: Nature. 2023 Jul 19;619(7970):572–84. doi: 10.1038/s41586-023-05915-x (PMC10356619; doi:10.1038/s41586-023-05915-x)
Supplement: Supplementary file 3 — Supplementary Figs. 1–12 and their figure legends. [file 41586_2023_5915_MOESM3_ESM.docx]

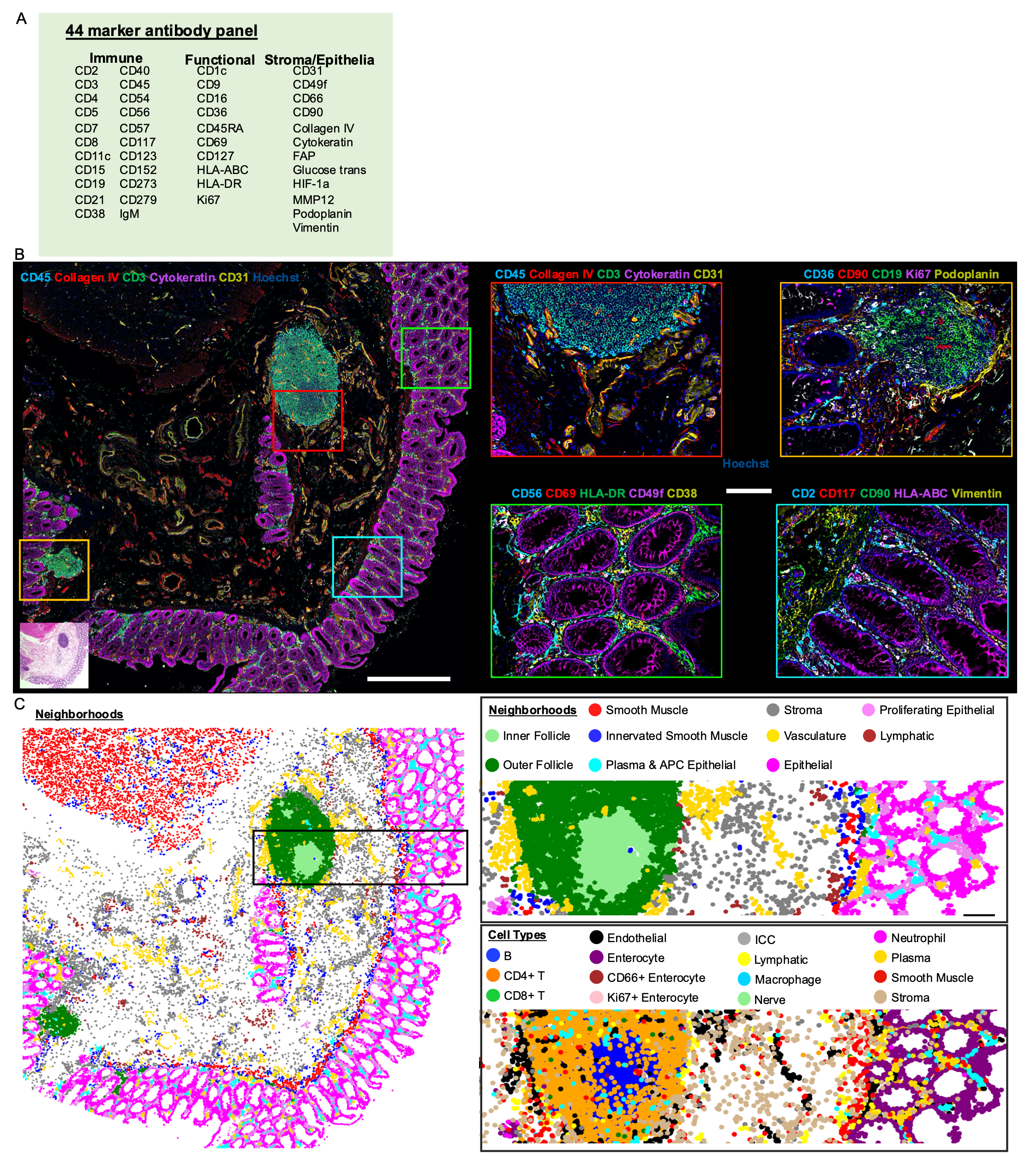


**Supplemental Figure 1:** CODEX multiplexed imaging of first donor B001 for small intestine and colon samples. A) 44 marker antibody base panel used for imaging. B) CODEX imaging of one of the 8 different sites taken from the colon for B001, with 5/44 markers shown (scale bar = 500 µm). Four zoomed in regions (scale bar = 100 µm) with various 5 color combinations of markers from the larger image (denoted by colored outline) with also an H&E image shown for the tissue. C) Multicellular neighborhood map for the same region with both cell type and neighborhood maps zoomed in around immune follicle regions. Representative image taken here is one of 8 taken from donor B001.

**
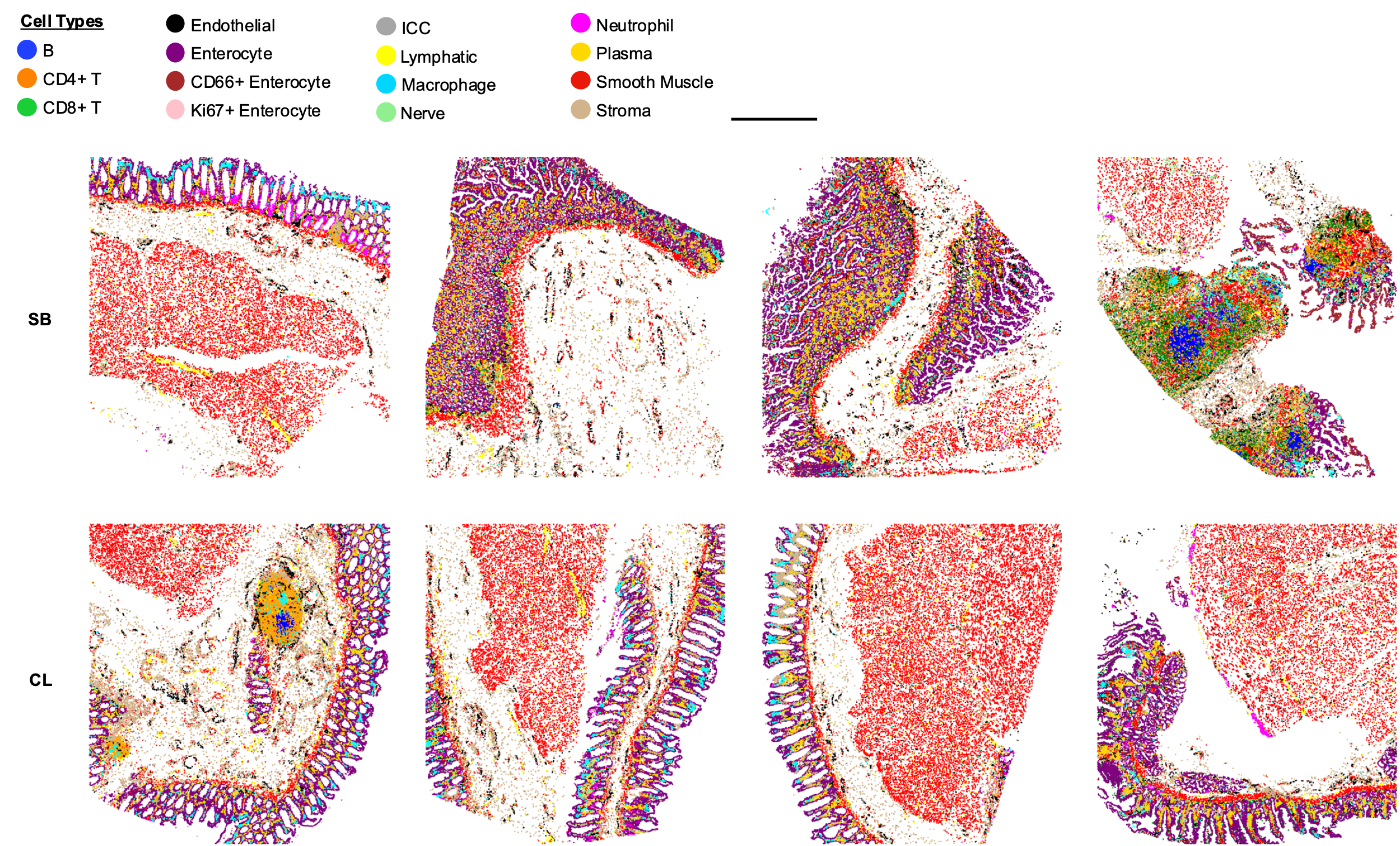
**

**Supplemental Figure 2:** Cell type maps for all 8 regions imaged. For donor 1 (B001) of the small intestine and colon (scale bar = 1 mm).


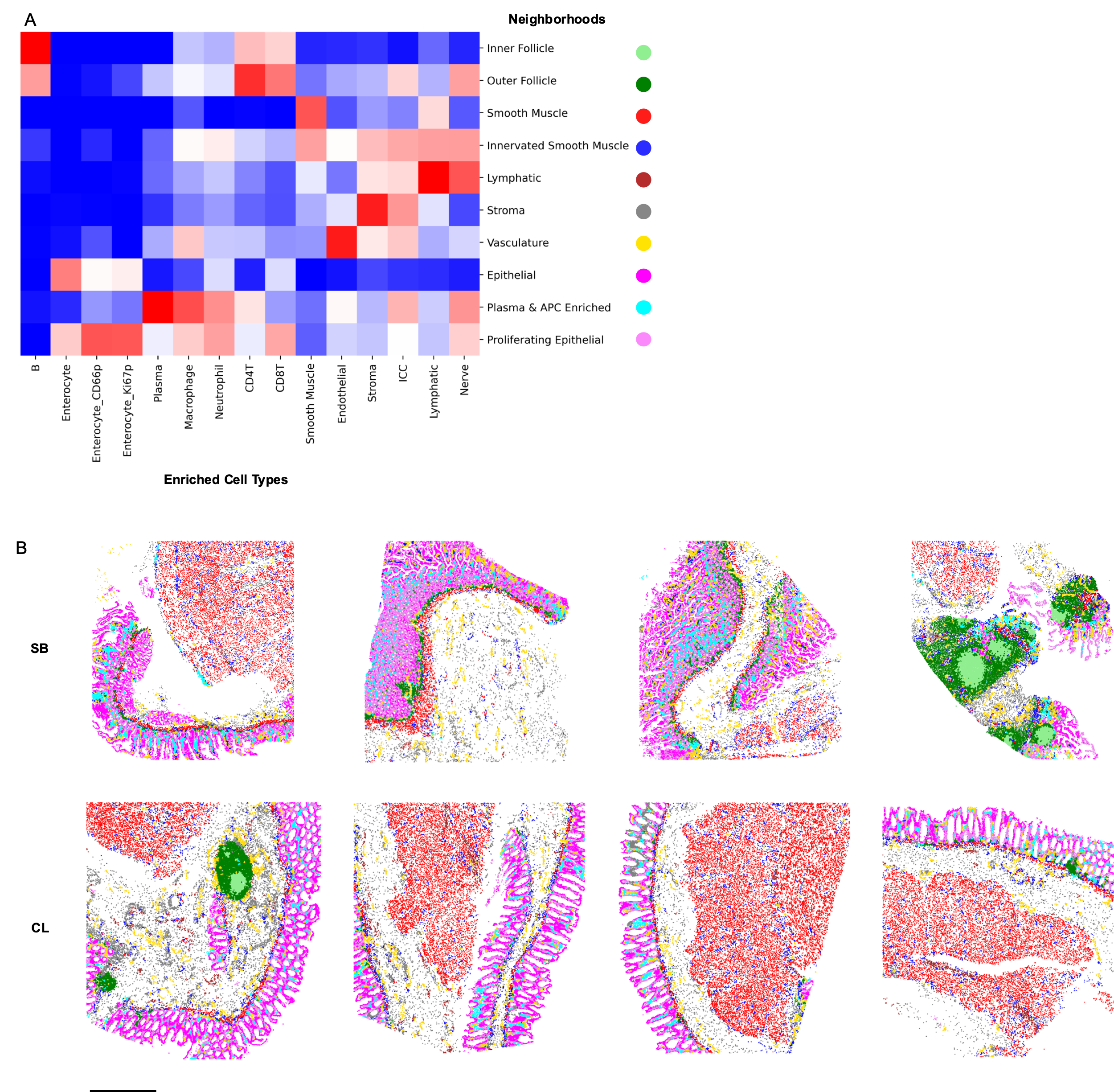


**Supplemental Figure 3:** Determination of multicellular neighborhoods in CODEX multiplexed imaging for donor 1 across all 8 regions. A) Cell type enrichment score within each neighborhood as represented in the heatmap. B) Cell type mapped back to each individual sample imaged (scale bar = 1 mm).


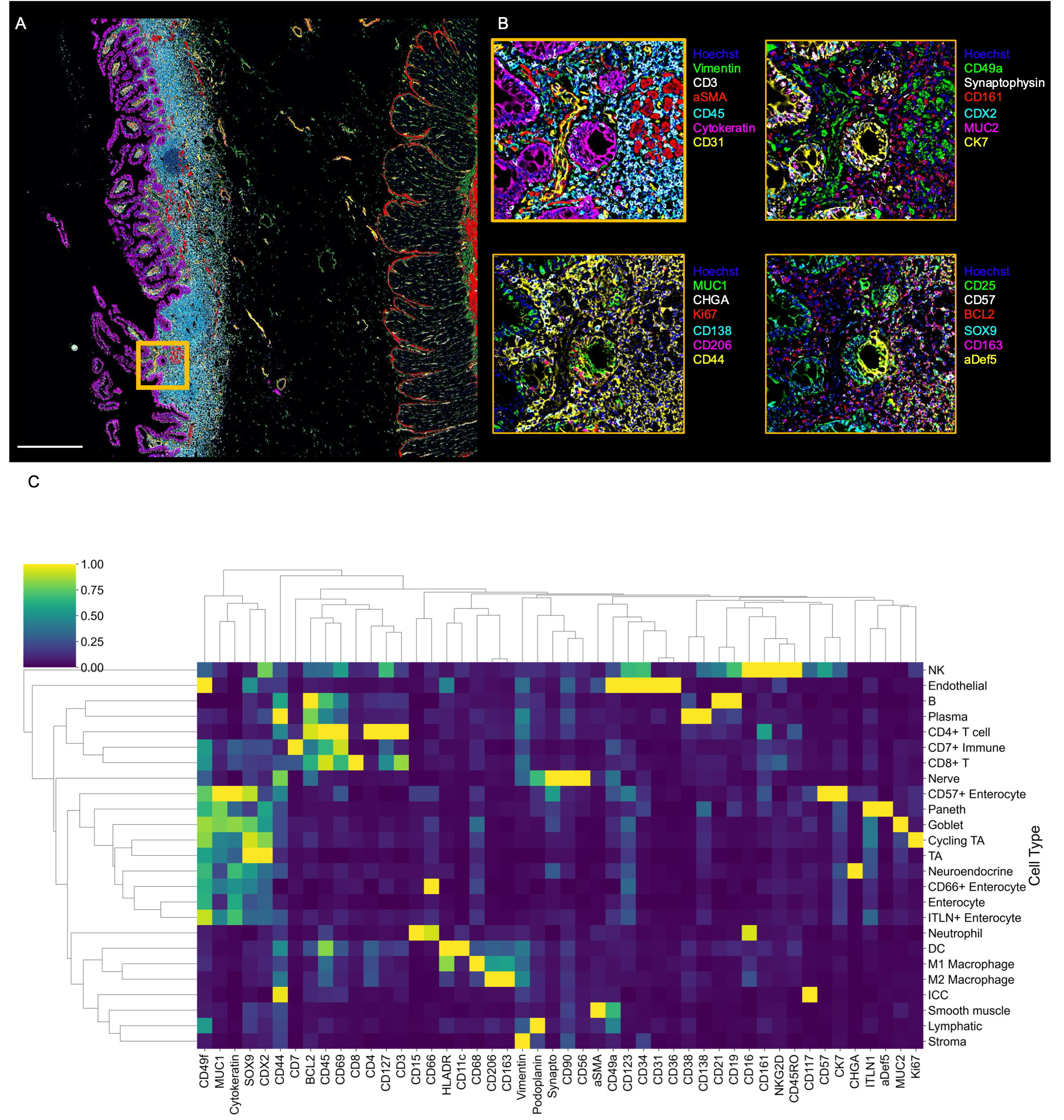


**Supplemental Figure 4:** CODEX multiplexed imaging with expanded 54 antibody CODEX panel. A) One region of B004 (total of 8) shown with 6 markers shown (Vimentin, CD3, aSMA, CD45, Cytokeratin, and CD31) (scale bar = 500 µm) B) Zoomed in regions with 6 markers each shown for the region highlighted (yellow box) in A with additional markers added to the panel highlighted (scale bar = 100 µm). C) Cell type by marker heatmap normalized both by column and row to show markers which define cell types from CODEX multiplexed imaging for samples B004,5,6.


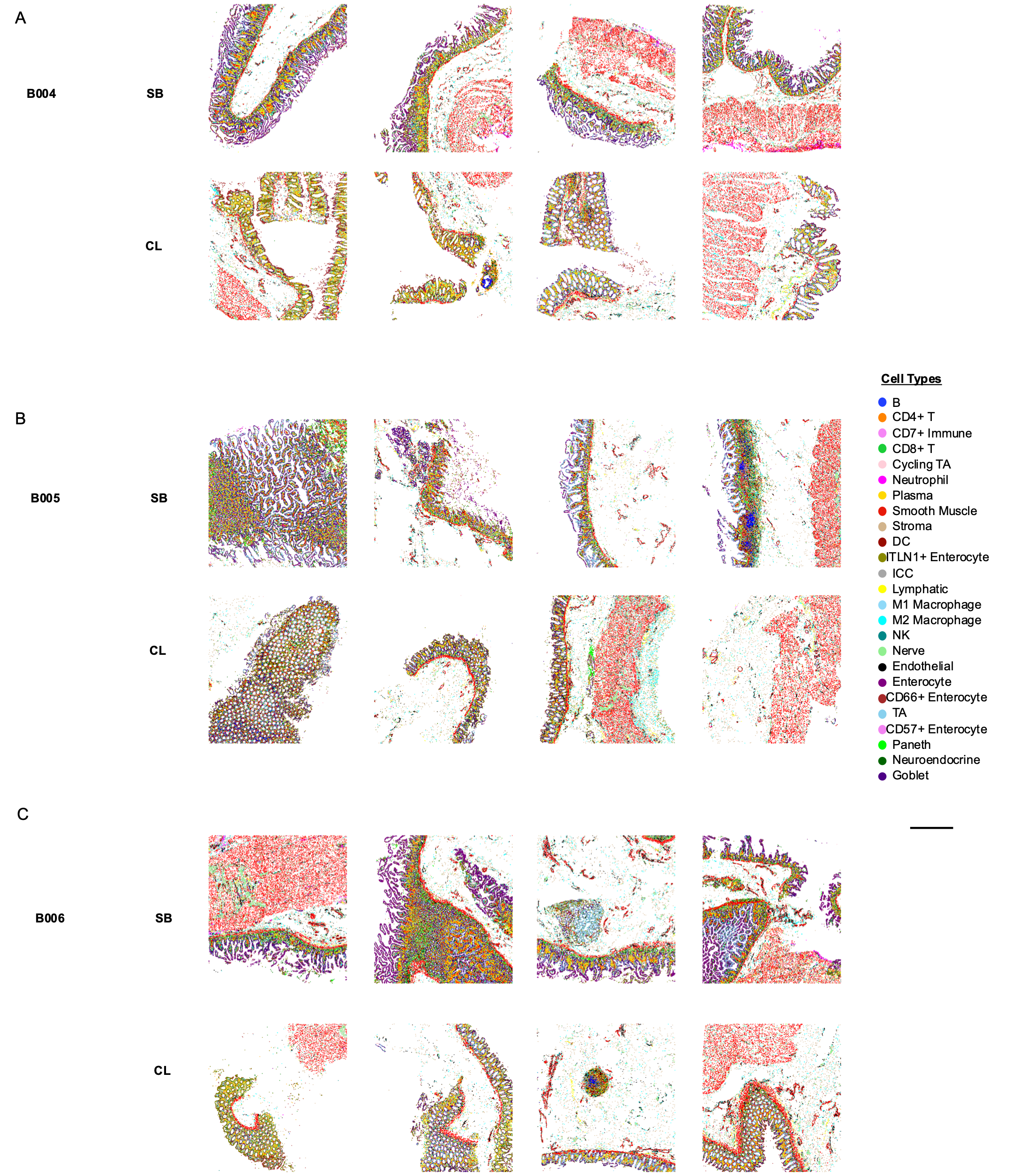


**Supplemental Figure 5:** Cell type maps for all 8 regions imaged. For donors A) B004, B) B005, and C) B006 of the small intestine and colon (scale bar = 1 mm).

**
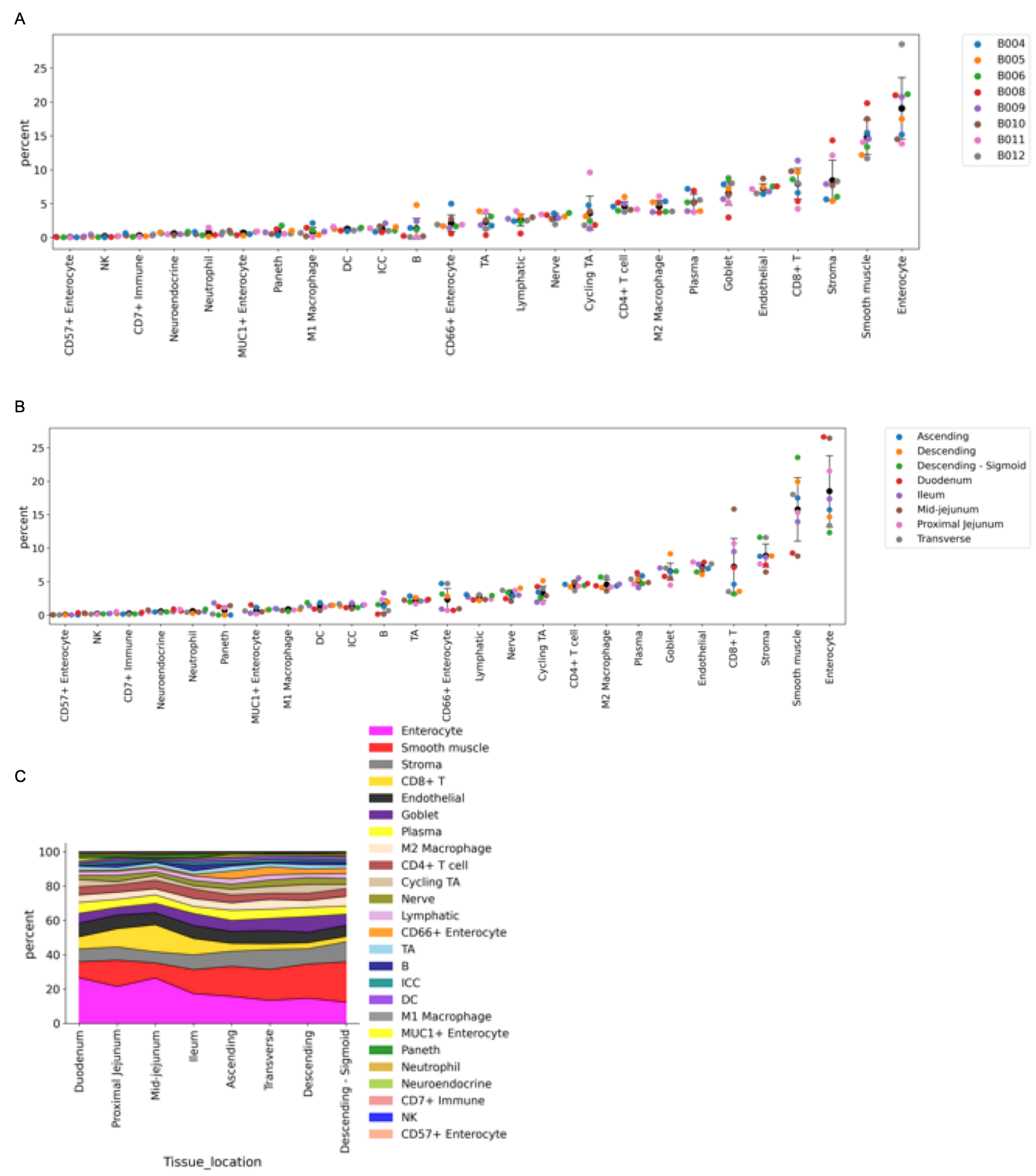
**

**Supplemental Figure 6:** Quantification of cell type percentages for all donors (n=8, error bars indicate standard deviation). A) Cell type percentage separated by donor. B) Cell type percentage grouped by location in the intestine. C) Cell type percent summation graph by location in the intestine with all cell types shown.


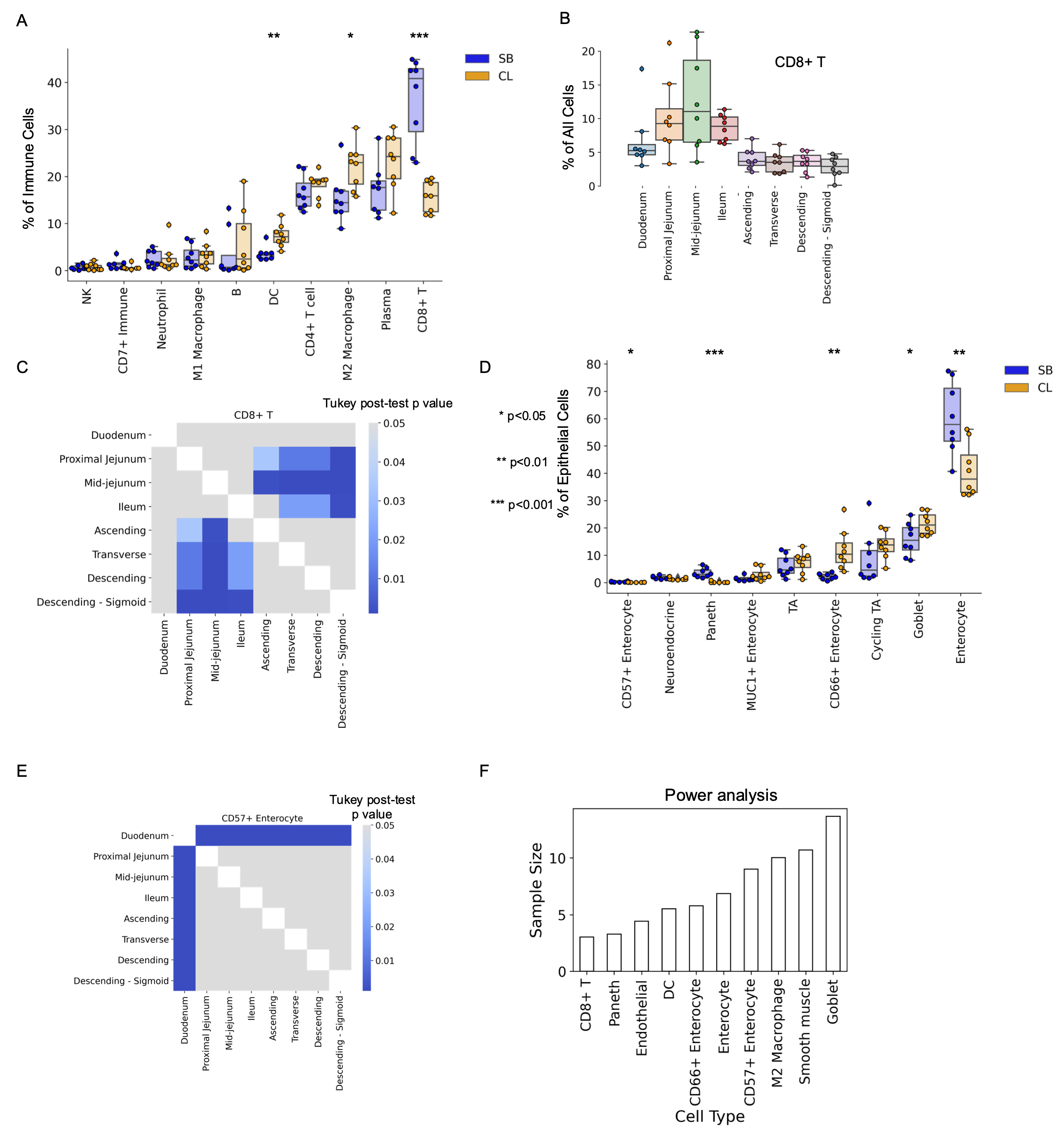


**Supplemental Figure 7:** Cell type percentages as determined by CODEX multiplexed imaging. A) Immune cell type percentages as a percent of all immune cells. B) Percentage of CD8+ T cells of all cell types across different areas samples from small intestine to colon. C) Heatmap of p values from Tukey post-test after a significant difference was found using a one-way ANOVA for the percent of CD8+ T cells across different areas of the small intestine and colon. D) Epithelial cell type percentages as a percent of all epithelial cells. E) Heatmap of p values from Tukey post-test after a significant difference was found using a one-way ANOVA for the percent of CD8+ T cells across different areas of the small intestine and colon. F) Power analysis for cell types that were significantly different from the small intestine to the colon using a power of 0.8 and alpha of 0.05. (* p value< 0.05, ** p value< 0.01, *** p value < 0.001, n=8 donors, by two-sided T test). All boxplots in figures are plotted as minimum, 25 percentile, median, 75 percentile, maximum, and outliers as points outside 1.5 the interquartile range.

**
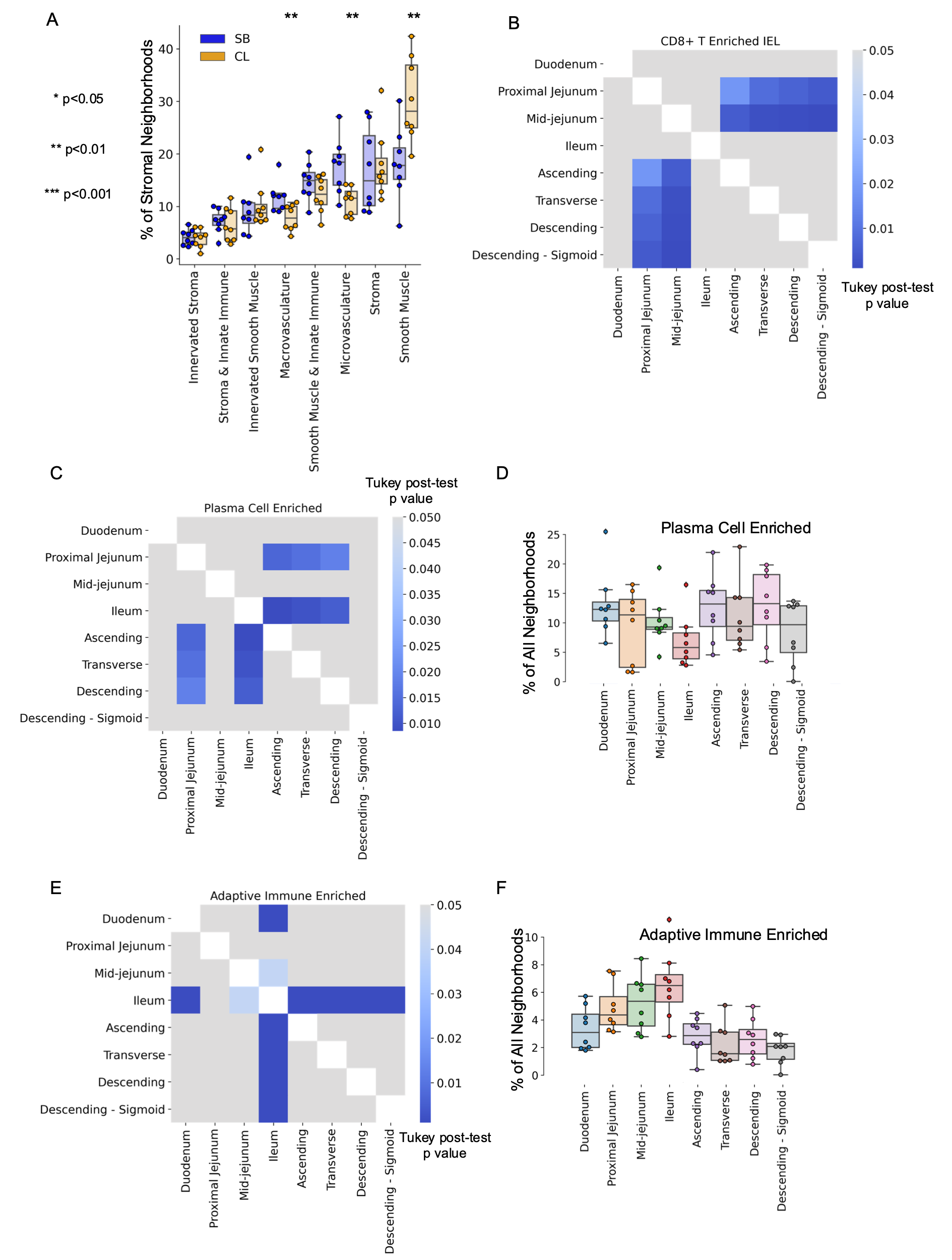
**

**Supplemental Figure 8:** Multicellular neighborhood percentage analysis from CODEX multiplexed imaging data of the intestine. A) Stromal multicellular neighborhood percentages as a percent of all stromal neighborhoods. B-C) Heatmap of p values from Tukey post-test after a significant difference was found using a one-way ANOVA for the percent of B) *CD8+ T cell IEL* neighborhood and C) *Plasma Cell Enriched* neighborhood across different areas of the small intestine and colon. D) Percentage of *Plasma Cell Enriched* neighborhood of all neighborhoods across different areas samples from small intestine to colon. E) Heatmap of p values from Tukey post-test after a significant difference was found using a one-way ANOVA for the percent of *Adaptive Immune Enriched* neighborhood. F) Percentage of *Adaptive Immune Enriched* neighborhood of all neighborhoods across different areas samples from small intestine to colon. (* p value< 0.05, ** p value< 0.01, *** p value < 0.001 by two-sided T test, n=8 donors).


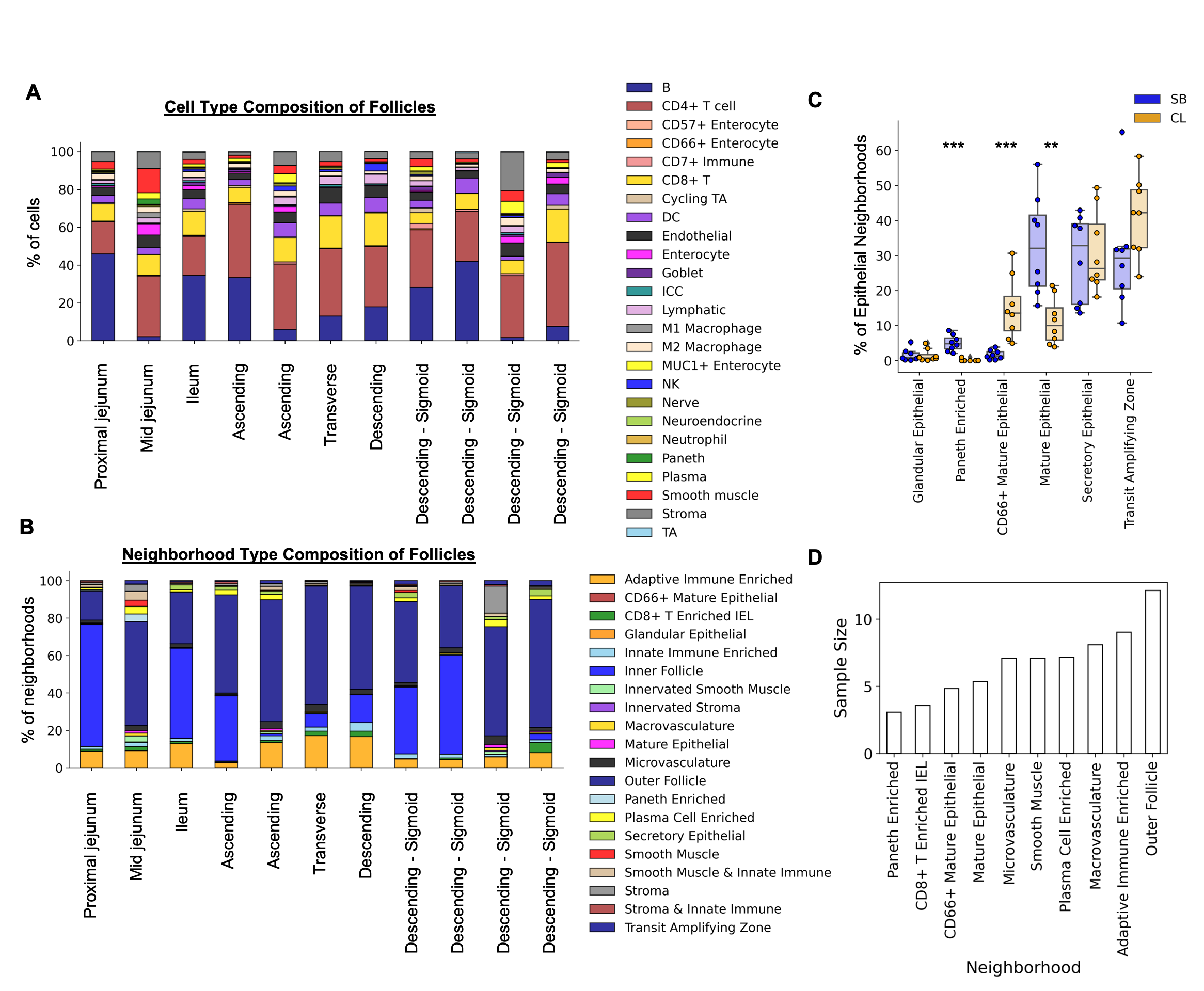


**Supplemental Figure 9:** Changes in neighborhood composition from the small bowel (SB) to colon (CL). A) Cell type percentage and B) multicellular neighborhood percentage for each follicle region segmented out from individual sections (11/64) imaged by CODEX multiplexed imaging. C) Epithelial neighborhood percentages as a percent of epithelial neighborhoods. D) Power analysis for multicellular neighborhoods that were significantly different from the small intestine to the colon using a power of 0.8 and alpha of 0.05. (* p value< 0.05, ** p value< 0.01, *** p value < 0.001 by two-sided T test, n=8 donors).


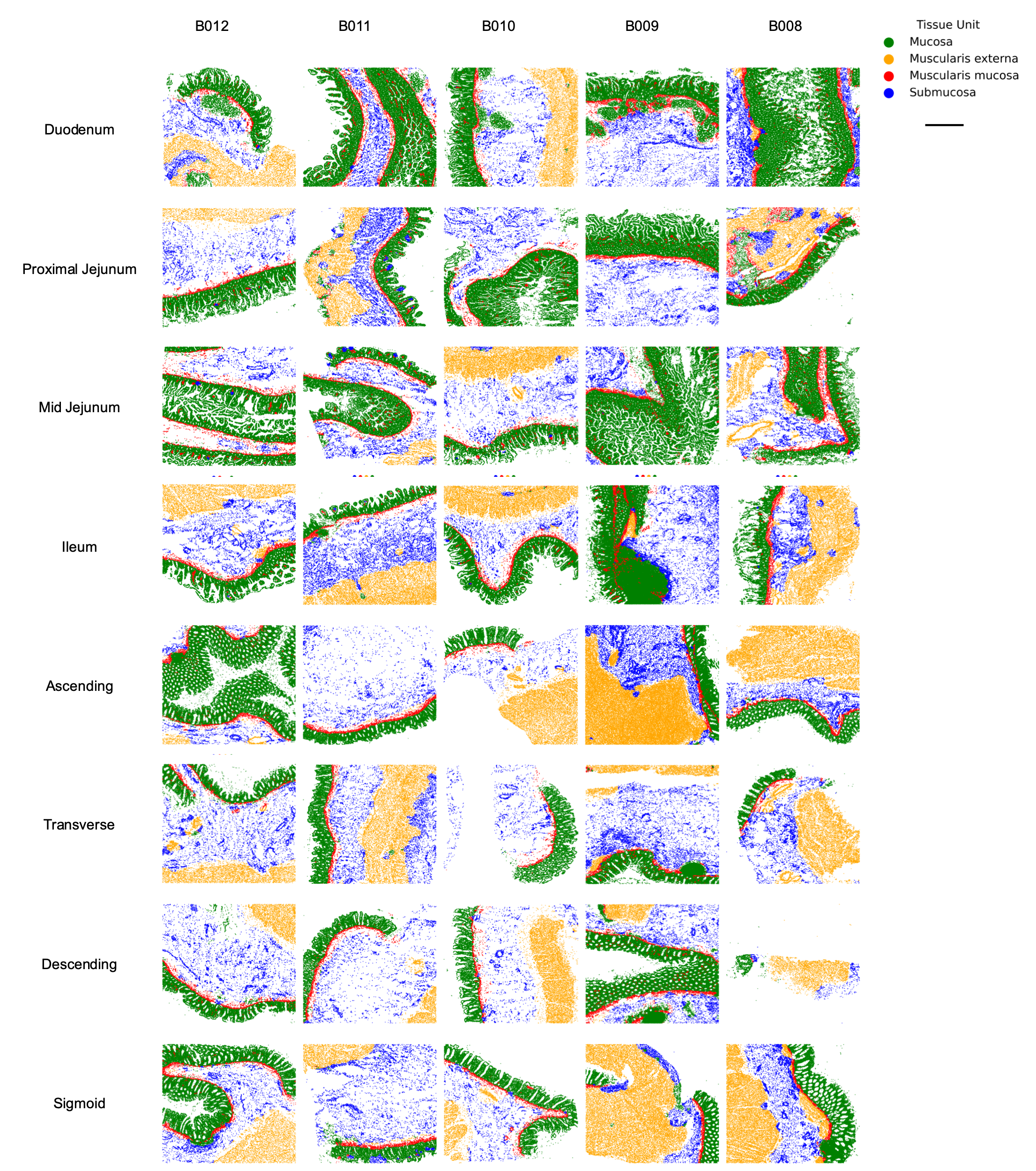


**Supplemental Figure 10:** Overall tissue unit represented on all 8 sections. From donors B008-B012 (scale bar = 100 µm).


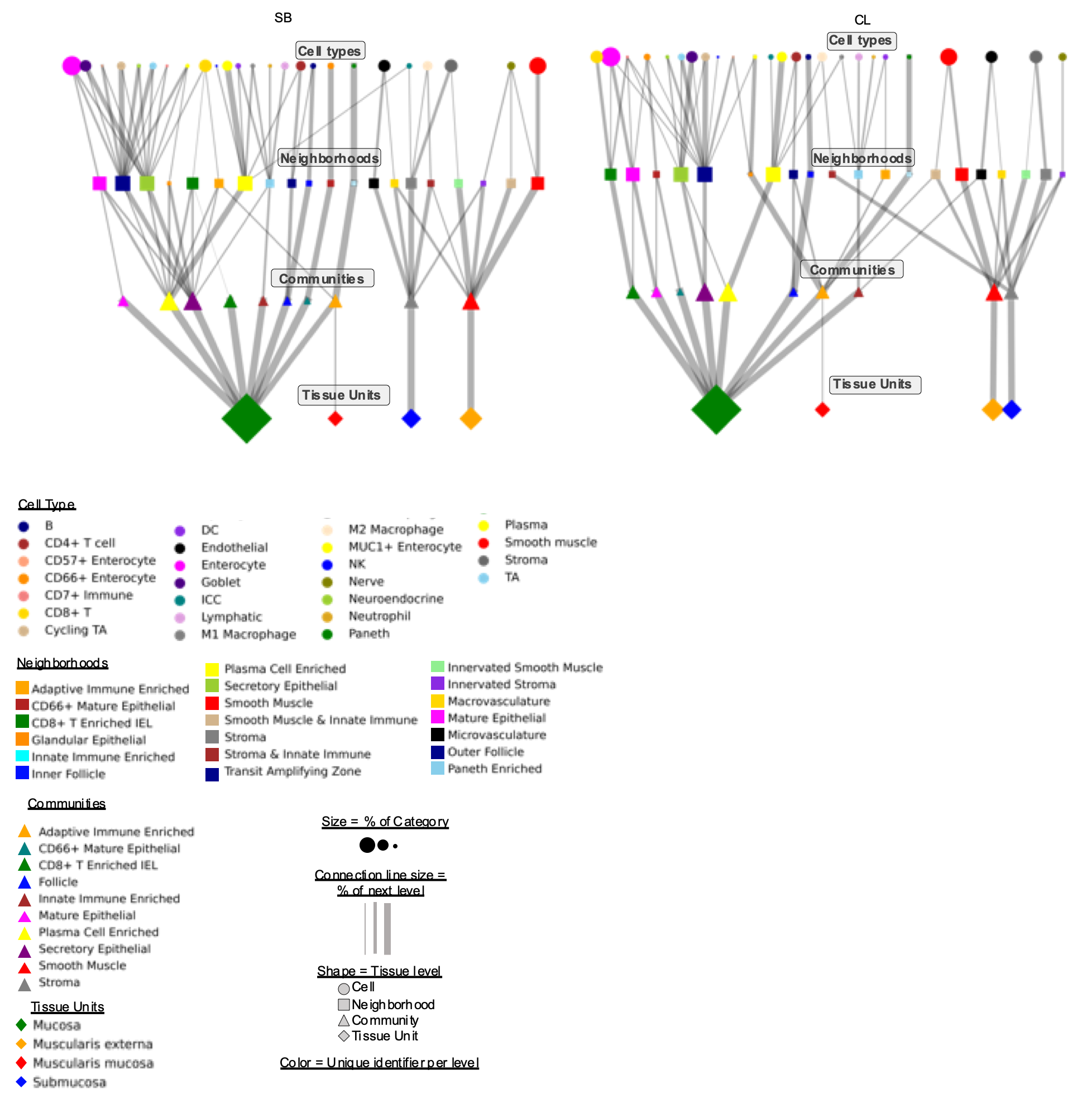


**Supplemental Figure 11:** Graph of multi-level structure of the tissue as broken down by the different structures. Shapes correspond to structural level, colors represent individual categories, size of shapes represents the percent contribution to tissue, and the size of connected lines represents the overall contribution to the next level of structure as moving down the graph in increasing tissue structural hierarchy. These are separated either by small bowel (SB) or colon (CL).


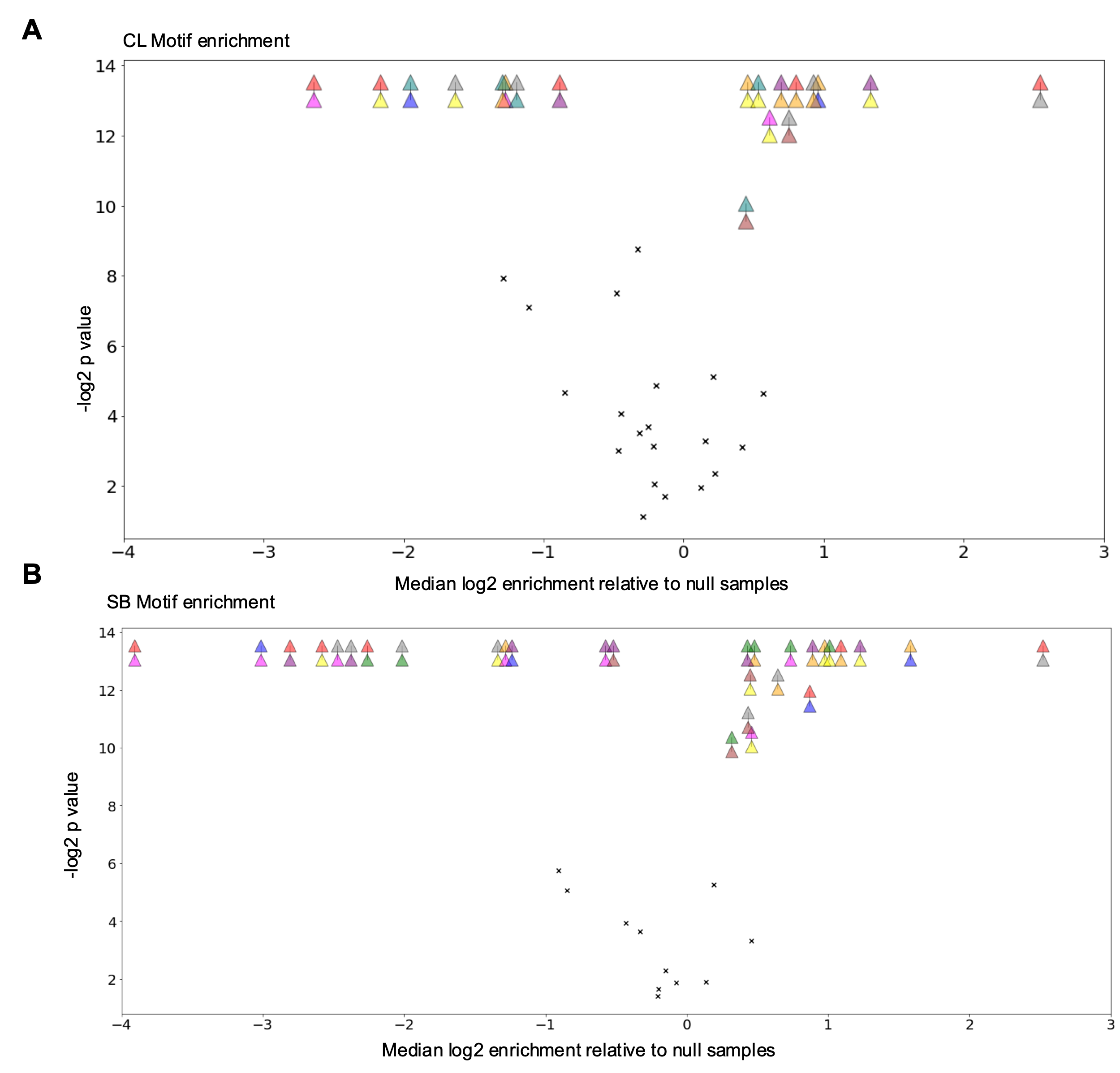


**Supplemental Figure 12:** Multi-community interaction motifs highlight organization of communities within the intestine. A-B) Community-community motifs that are significantly enriched as compared to a null distribution of motif instances created from random permutation of tissue graph labels, where A) shows motifs that are significant in the colon and B) shows motifs that are significant in the small bowel (p values were Bonferroni corrected by multiplying by twice the number of tests conducted in each comparison group).
